# Effect of Pb-Contaminated Water on *Ludwigia stolonifera* (Guill. & Perr.) P.H. Raven Physiology and Phytoremediation Performance

**DOI:** 10.3390/plants11050636

**Published:** 2022-02-26

**Authors:** Amany Aboelkassem, Nurah M. Alzamel, Mashail Nasser Alzain, Naglaa Loutfy

**Affiliations:** 1 Botany and Microbiology Department, Faculty of Science, Sohag Univerisity, Sohag 82524, Egypt; 2 Department of Biology, College of Sciences and Humanities, Shaqra University, Shaqra 11961, Saudi Arabia; nalzamel@su.edu.sa; 3 Department of Biology, College of Sciences, Princess Nourah Bint Abdulrahman University, Riyadh 11451, Saudi Arabia; mnalzain@pnu.edu.sa; 4 Botany and Microbiology Department, Faculty of Science, South Valley University, Qena 83523, Egypt

**Keywords:** bioaccumulation, toxicity, antioxidant enzymes, aquatic plants, photosynthetic pigments

## Abstract

A laboratory experiment was led to examine the lead bioaccumulation capacity of *Ludwigia stolonifera* (Guill. & Perr.) exposed to various Pb concentrations (0, 10, 25, 50, and 100 mg/L) for 1, 3, 5, and 7 days. The lead accumulation increased as the metal concentrations in the solution increased and over time, to an extreme accretion of 6840 mg/kg DW(dry weight) at 100 mg/L of lead on the 10 days exposure. The proportion removal efficiency, translocation factor, and bioconcentration factor of the plant were assessed. The maximum bioconcentration factor values (1981.13) indicate that the plant was a Pb hyperaccumulator, and translocation factor values (1.85), which are >1, indicate fit of *L. stolonifera* for eliminating Pb in Pb-contaminated water. Photosynthetic pigments were decreased with increase of Pb concentration and time exposure. Total chlorophyll content and Chl a/b ratio lowered to between 46 and 62% at 100 mg/L Pb after 10 days exposure. Protein content and soluble carbohydrate indicated a similar trend, which showed the highest decrease (7.26 and 36.2 mg/g FW(fresh weight), respectively) at 100 mg/L of Pb after 10 days. The activity of the antioxidant enzymes superoxide dismutase, ascorbate, and peroxidase was increased significantly in comparison to the control. The results indicate that *L. stolonifera* is a newly recognized Pb hyperaccumulator (6840 mg/kg DW), but physiological status indicates that the plant is not tolerant to high Pb concentrations.

## 1. Introduction

Toxic metals contamination in the river environment has converted a main reason of worry for some developing nations due to lack of mitigation measures and high cost of remediation. Toxic metals may reach surface and groundwater streams through numerous transport routes after being introduced into the environment. Industrial effluents comprising toxic and hazardous wastes are dumped into the ecosystem in some locations, causing environmental harm [1,2].

Toxic metals are known to be non-biodegradable and to persist for a long time in both aquatic and terrestrial environments [3]. Lead (Pb), a hazardous metal extensively employed in industrial activity, is found in both terrestrial and aquatic habitats [4,5]. Lead (Pb) accumulates in the human body and causes major issues. Pb has been linked to functional difficulties in the kidneys, joints, cardiovascular system, and reproductive system in humans. Pb is also dangerous to plants in the same way that it is to humans. Lead accumulation in plants boundaries photosynthesis and enzyme function and disrupts the water and nutrient balance, resulting in stunted growth and chlorosis [6].

Phytoremediation is a wide term for the removal or decomposition of pollutants from soil or water by using plants [7,8,9]. One of the most hopeful choices for eliminating organic and inorganic contaminants from soil and water is phytoremediation. Because of its performance, inherent green character, end-use of by-products, and overall economic effectiveness, this method is well embraced by the public [10,11,12]. Aquatic macrophytes have proven to be one of the applicants in the aquatic system for pollutant uptake and biological indicators of toxic metal [5,13,14]. High capacity to collect hazardous metals, high biomass output and quick growth, and bioconcentration factor (BCF) and translocation factor (TF) values greater than one portray an ideal hyperaccumulator plant [15,16].

Many metal-hyperaccumulator plants have been investigated in the past, but further research is needed to confirm their phytoremediation effectiveness. Plants’ ability to accumulate pollutants such as harmful metals and chemical compounds is now being investigated by scientists. The present study is focused on an invasive macrophyte (*Ludwigia stolonifera*) with a rapid rate of development and propagation that poses a threat to local ecosystems. *L. stolonifera* has the ability to act as a phytoremediator, removing various contaminants from aquatic ecosystems [17,18]. Under laboratory conditions, the current study’s goal was to assess *L. stolonifera’s* Pb uptake and phytoremediation capacity.

## 2. Results

### 2.1. Lead Removal and Accumulation

The different concentrations of lead in the medium were significantly different over ten days (Table 1). On the tenth day of exposure, the maximum removal efficiencies were 95.58, 92.61, 91.71, and 90.08%, when *L. Stolonifera* was exposed to 10, 25, 50, and 75 mg/L of lead, respectively (Table 2). The bioaccumulation rate of lead increased with the increase in the time of exposure (Table 3). It is clear that the significant increase in lead concentration occurs 24 h after exposure (320. 33, 866.66, 1871.75, and 3963.3 mg/kg DW for 10, 25, 50, and 100 mg/L of Pb treatment, respectively) and then continues to increase but in a less severe manner, reaching maximum mean values of 875, 2681.67, 4975, and 6840 mg/kg DW at 10, 25, 50, and 100 mg/L of treatment with lead, respectively, at the end of the experiment.

Table 4 exhibits that the maximum BCF value (1981.13) was obtained when treated with 10 mg/L of lead at the 10th day of the study. Likewise, the translocation factor (TF) value for *L. stolonifera* is 1.17, 1.16, 1.49, and 1.85 at 10, 25, 50, and 100 mg/L of treatment with lead, respectively at the end of experiment (Table 4).

### 2.2. Pigment Content

**Figure 1:**
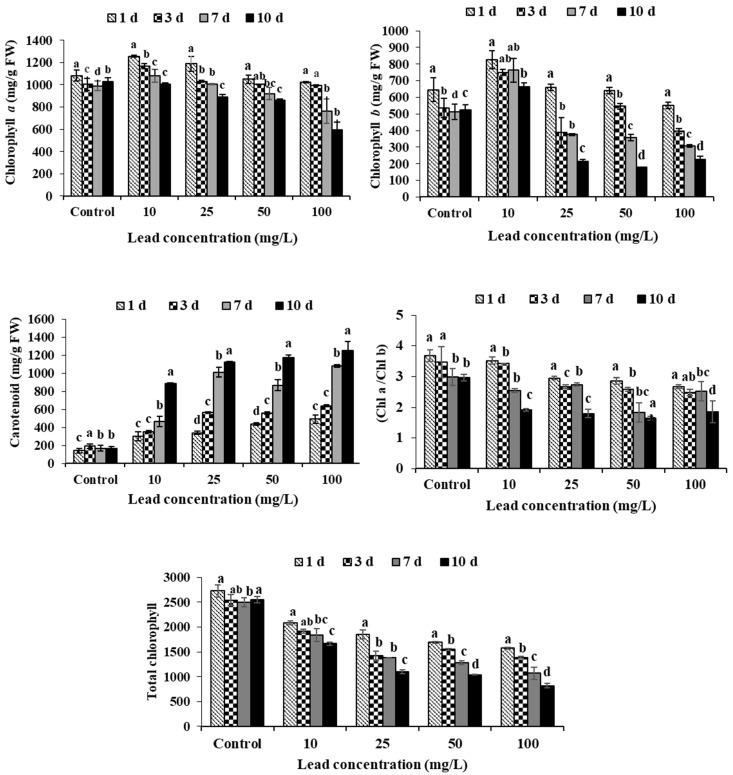
Effects of lead toxicity on photosynthetic pigments content in *Ludwigia stolonifera*. Means values ± SD (*n* = 3). ANOVA was significant at *p* < 0.05. Different letters indicate significantly different values for a particular treatment group (Tukey’s *p* < 0.05).

Pb has an effect on the concentration of photosynthetic pigments in *L. stolonifera*, as shown in Figure 1. During different treatments and exposure days, there was a significant decrease (*p* < 0.05) in photosynthetic pigments of *L. stolonifera* relative to their respective controls. Compared to the control, the lowest content of chlorophyll a and b in the Pb applications was found at 100 mg/L Pb (57.23%) after 7 days and 50 mg/L Pb (33.82%) after 5 days (Figure 1) respectively. At varied concentrations of Pb (Figure 1), total chlorophyll content and Chl a/b ratio showed a similar trend, which was initially lowered to between 46 and 62% at 100 mg/L Pb after 10 days of exposure. On a 10-day exposure, the reductions were more pronounced. Unlike chlorophyll levels, increases in carotenoids levels in Pb treatments (10, 25, 50, and 100 mg/L) were observed during the sampling period as compared to their respective controls (Figure 1).

### 2.3. Antioxidant Enzymes

**Figure 2:**
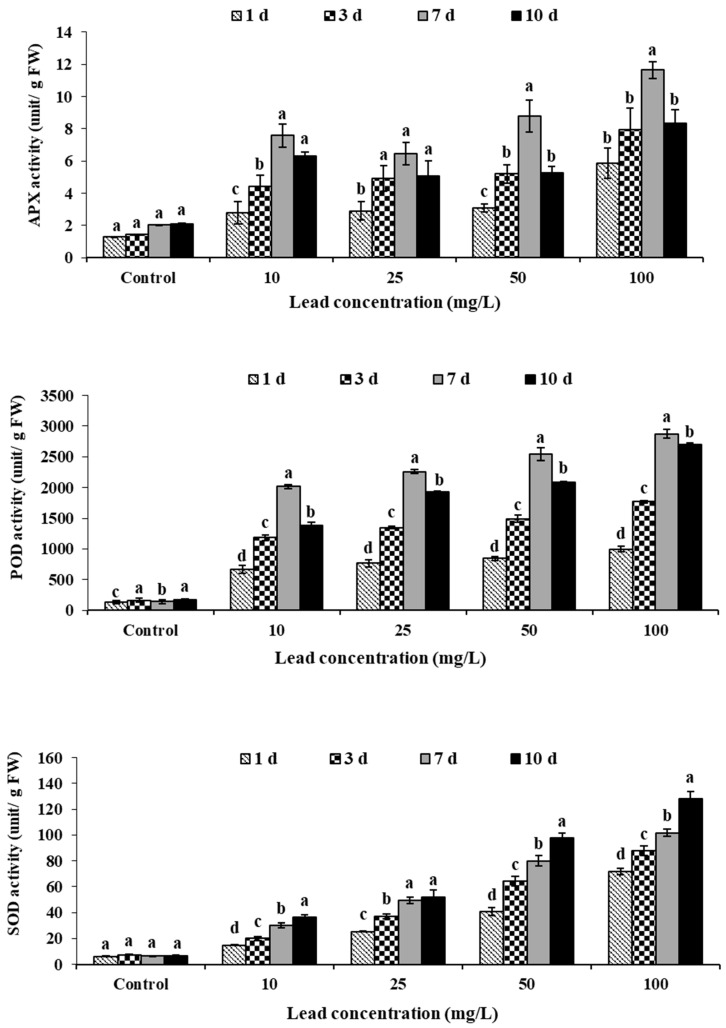
Effects of lead toxicity on APX content, POD, and on SOD activities in *Ludwigia stolonifera*. Mean values ± SD (*n* = 3). ANOVA was significant at *p* < 0.05. Different letters indicate significantly different values for a particular treatment group (Tukey’s *p* < 0.05).

During the exposure period, the enzyme activity in the leaves increased significantly in comparison to the control (Figure 2). On day 7, the maximum APX activity in plants exposed to Pb was determined to be 11.648 unit/g FW at 100 mg/L (57.37 percent higher than the control). Exposure duration (r = 0.50, *p* < 0.001) and exposure concentration (r = 0.55, *p* < 0.001) had a significant effect on APX activity in plants exposed to Pb. POD activity improved significantly with the extension of treatment time (r = 0.75, *p* < 0.001) and increasing concentration (r = 0.41, *p* < 0.01) under the influence of lead stress (Table 5). The overall POD activity was 7.6 times, 10.8 times, and 20.2 times that of the control value on days 1, 3, and 7. POD activity, on the other hand, decreased to 16.3 times that of the control after a 10-day exposure (Figure 2). Table 5 demonstrates a strong positive correlation between POD activity and lead stress, with POD activity significantly higher in lead-exposed plants than in controls. Lead exposure affected SOD activity (Figure 2), which increased significantly with treatment time (r = 0.47, *p* < 0.01) and lead concentration (r = 0.84, *p* < 0.001). From days 3 to 10, plants treated with lead concentrations of 100 mg/L showed a rapid rise, while other concentrations showed a steady increase. On day 10, the maximum activity was observed at 100 mg/L, which was 128.1 percent higher than the control; however, significant increases were observed for all other concentrations.

### 2.4. Fresh and Dry Weight

**Figure 3:**
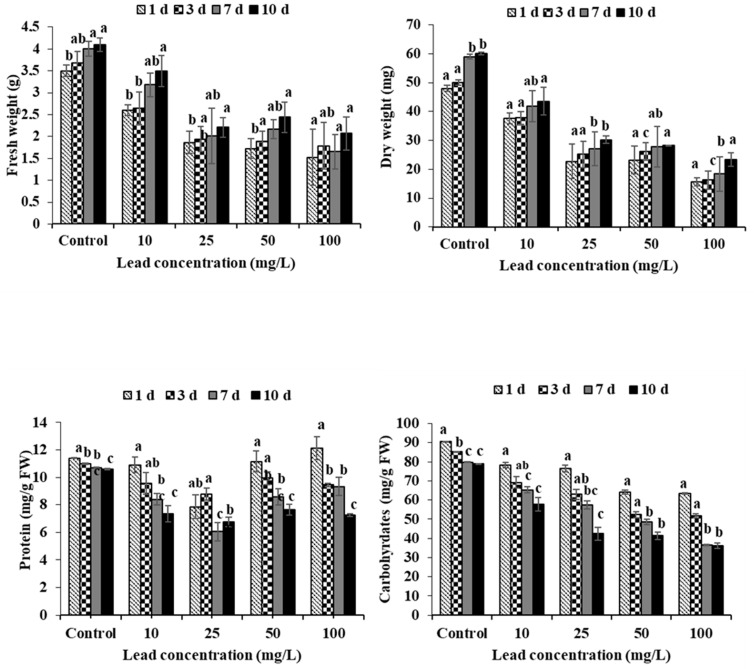
Effects of lead toxicity on fresh weight (g), dry weight (mg), protein content, and soluble carbohydrate concentration of *Ludwigia stolonifera* at different concentration and exposure times. Mean values ± SD (*n* = 3). ANOVA was significant at *p* < 0.05. Different letters indicate significantly different values for a particular treatment group (Tukey’s *p* < 0.05).

Figure 3 shows the fresh and dry weights of the plants affected by Pb at various times and concentrations. After 7 days of Pb application, the maximum reduction in fresh mass was 41.3 percent less than that of the controls at a concentration of 100 mg/L (Figure 3). After 10 days, the highest dry weights in the Pb applications compared to the control were obtained at 10 mg/L (72 %). The lowest dry weights for Pb groups, on the other hand, were recorded at 100 mg/L 41.5 % after 1 day. While reductions in dry weights were generally determined depending on the increase in Pb concentration (*p* < 0.001, r = −0.76) (Table 2).

### 2.5. Protein

Protein contents of *L. stoloniferia* exposed to Pb are presented in Figure 3, which were statistically significant (*p* < 0.001). Protein contents were significantly influenced by both exposure time (r = −0.73, *p* < 0.001) and metal concentration (r = 0.27, *p* < 0.01) (Table 5). The highest decrease in protein content was 7.26 mg/g FW observed at 100 mg/L of Pb after 10 days.

### 2.6. Carbohydrate

Effects of lead toxicity on the soluble carbohydrate content, which were measured as indicators of growth status and lead stress, are shown in Figure 3. A decreasing tendency in carbohydrate amount was observed as Pb concentration increased during 10 days of exposure. Notably, reductions in carbohydrate content were observed at high concentrations of lead and with long exposure times. Furthermore, the maximum reduction in carbohydrate content was 45.8% less than that of the controls at 10 days.

## 3. Discussion

The phytoremediation potential of many hyperaccumulator plant species is limited by their sluggish development rate, low biomass, and typically close association with a specific ecosystem [8]. As a result, the rapid development of heavy metal resistant plants for phytoremediation has become critical. Plant tissue cultures are one of the most efficient methods for producing these plants in large quantities quickly and repeatedly. The buildup of heavy metals is known to be a time- and concentration-dependent process [5]. The results of this study showed gradual removal of lead in the different concentrations of water samples as indicated by their respective percent remaining. The results reported that lead removal efficiency (RE) increased with increasing exposure time and concentration. Comparable results have been obtained by Abd-Elaal et al. [6] in *Echinochloa* pyramidalis and *Ludwigia stolonifera* with removal percentages of heavy metals (Pb, Cd, and Ni) between 77 and 95% in 10-day incubation period. This may be attributed to the fact that most of the active sites are occupied by toxic metal from surrounding media [19]. In this study, an increase of metal removal and accumulation was observed in a time dependent manner. These findings are consistent with those of Aboelkassem et al. [5], who found that the studied plants are Pb tolerant and can withstand harsh conditions. The relationship of Pb BCF to Pb exposure concentration was negatively correlated. This may be because, while lead concentrations rise over time, internal lead accumulation does not keep pace with levels of external exposure. This suggests that plants have evolved a unique mechanism to effectively regulate the deposition of lead. As a result, the BCF values decrease as the Pb exposure concentration in the medium rises. The BCF value of *L. Stolonifera* was greater than 1000 in the recent study, indicating that it could be considered a strong lead accumulator. The Pb BCF has also been found in the following species: *Najas indica* [20], *Pistia stratiotes* [21], *Acorus gramineus* [22], *Ludwigia peploides* [23], and *Echinochloa pyramidalis* [6]. Similarly, TF values greater than one suggest that the plant has substantial metal translocation pathways to the leaves [22,24,25,26].

The chlorophyll pigment is the most important photosynthetic pigment for light absorption and energy transfer [27]. Several studies have found changes in photosynthetic pigments in plants exposed to toxic metals, with the results suggesting that Cd and Pb decreased photosynthetic development due to increased ROS (reactive oxygen species) generation [28,29,30,31,32]. Since the relationship between mineral concentration and chlorophyll content is inverse, growth inhibition and low levels of chlorophyll present in the leaves of plants exposed to lead can also be due to a lack of essential nutrients needed for physiological activities, as shown by the negative correlation coefficient in Table 5. Besides, it was detailed that chlorophyll a/b value demonstrated the interconversion of chlorophyll a and chlorophyll b, called the chlorophyll cycle, which was fundamental to the forms of greening, light acclimation, and senescence [33]. As displayed in Figure 1, compared with control, chlorophyll a/b diminished altogether (*p* < 0.001); in any case, statistically significant contrasts were observed among the four toxin concentration treatments (*p* < 0.001). Huan Xiao et al. [34] detailed that the diminished chlorophyll a/b value may be considered as the change chlorophyll a to chlorophyll b, and the control can be a protective physiological reaction for the plants of *L. salicaria* L., *Nymphaea L.,* and *Vallisneria. natans* (Lour.) Hara to pollutants. Lead increased the content of carotenoids in the leaves of the plants tested. Carotenoids protect chlorophylls from free radical formation [35,36] and are thought to play a role in chlorophyll defense under stress conditions by quenching photodynamic reactions, replacing peroxidation, and collapsing membranes in chloroplasts [37].

Drought, intensity of illumination, low or high temperature, salinity, UV radiation, ozone, and heavy metals are all stimuli that the antioxidant system can use to decide a plant’s resistance and tolerance too [19]. To eliminate ROS, plants have a system of enzymatic and non-enzymatic antioxidants [38], with the antioxidant enzymes superoxide dismutase (SOD), catalase (CAT), and peroxidase (POD) playing crucial roles [5,16]. The result of this study indicates that oxidative stress is caused by Pb stress at various stages of treatment. The plants were also placed under anaerobic stress as a result of the prolonged water inundation. The overproduction of ROS and the induction of responses of the main antioxidative enzymes were caused by these negative factors. At exposure time, APX activity did not change significantly in the 10–25 mg/L concentration range, indicating that the plants had reached the limits of their ability to scavenge oxygen free radicals using APX. Increased SOD activity reflected both a defensive mechanism and a sign of heavy metal stress in the plants in this investigation when the lead content was relatively low. Only at higher doses (50–100 mg/L) and for longer periods (7and 10 days) was a robust SOD response to lead stress found, showing the plants’ great resistance to lead. Salawu et al. [23] discovered that the activity of SOD in *Ludwigia peploide* leaves cultivated with chromium and zinc was inhibited within two weeks of cultivation, which may be due to high oxidative stress on the plant tissue, but that the plant was able to recover from the oxidative stress and the activity of SOD increased at a later stage of cultivation. High POD activity indicates the POD was unaffected by lead toxicity and played a critical role in oxidative stress defense. POD may function as a protection molecule by removing H_2_O_2_ when a plant is stressed and also catalyzes the generation of ROS when the damage is too extreme, aggravating membrane lipid peroxidation and causing additional damage [39,40,41].

The dry and fresh weights of heavy metal-exposed plants are critical parameters for determining toxicity symptoms. Heavy metal exposure at high levels reduces plant biomass and inhibits plant growth [42]. After 7 days in Pb applications, the concentration of 100 mg/L resulted in the greatest decrease in fresh mass compared to the control (41.3%). Reference [43] reported that *C. demersum* plants were exposed to Cd and Pb (1–100 mg/L) for 1–7 days, with 100 mg/L Pb causing the greatest decrease in fresh weight compared to the control. In the current study, there was a general decrease in dry mass with increasing Pb concentration when compared to the control. This decrease in dry weight could be caused by the degradation of biochemical parameters such as enzyme activity, protein anabolism, and photosynthetic activities due to Pb toxicity [44]. Likewise, as metal concentrations and exposure time increased, plant protein content decreased. Similarly, *C. demersum* [43,45] and *N. indica* [20] have previously reported decreases in protein content of *L. Stolonifera* under high-concentration metal stress. This decrease could be attributed to an increase in the total amount of ROS which could have harmed numerous proteins. It could be related to protease or other catabolic enzymes being activated by heavy metal toxicity [42]. When *L. Stolonifera* plants were exposed to a discharge of water contaminated with various concentrations of Pb on a daily basis for ten days, their leaves revealed a dramatic reduction of more than 45% in the content of soluble carbohydrates when compared to control plants. Dhir et al. [46] detailed that the nearness of toxic metals within the environment influences the generation of dissolvable carbohydrates in *Salvinia*, diminishing their photosynthetic capacity. Furthermore, the decrement in solvent carbohydrates in *S. biloba* leaves may be related with a Pb-induced closure of stomata that disables CO_2_ accessibility in a comparable way as detailed for *S. minima* [47]. These perceptions connect with the above-mentioned lessening in total chlorophyll content, particularly that of Chl a and of proportion between Chl a and Chl b, which are both vital parameters in appraising the impact of an environmental event in a plant [48].

## 4. Materials and Methods

### 4.1. Plant Material and Growth Conditions

*Ludwigia stolonifera* (Guill.&Perr.) was obtained from the mainstream of the River Nile bank, Sohag, Egypt and they transferred to the laboratory. Because of the lack of anthropogenic activity and the low levels of metals detected, preliminary measurements (Pb concentration < 25 mg/kg in bottom sediment) in this location were used as a contamination control site [49]. To eliminate clinging particles, the plants were rinsed numerous times with tap and distilled water. Plants with identical biomass and height were chosen and stored separately in 20 L aquariums with half strength Hoagland’s solution with a pH of 7 [50] for 15 days before acclimatization. Then, the acclimatized plants were transferred and maintained in aquariums with 5% Hoagland’s solution including varied concentrations of working Pb standard solutions 0, 10, 25, 50, and 100 mg/L. They were then exposed to Pb concentrations over a period of time (0, 1, 3, 7, and 10 days). Pb of analytical grade was provided; Pb(NO_3_)_2_ (Sigma, St. Louis, MO, USA) was used as the source of Pb. The trials were carried out in a controlled temperature (24 ± 2 °C by using XH-M452 Thermostat Temperature Humidity Control Sw) and with light (1950 Lux).

### 4.2. Metal Content Measurement

Water samples (5 mL) and plant (5 g fresh weight) were collected simultaneously as in the experimental design described above at 1, 3, 7, 10-day intervals. Super pure nitric acid (0.1 mL/15 mL sample) was used to acidify the collected water samples. The plants were gathered and washed with deionized water after each time interval to remove any metal that had adhered to their surface. The plant samples were dehydrated for 48 h in an oven at 85 °C, then separated into leaves, stems, and roots, crushed by mortar to facilitate digestion of the organic matter. After that, 0.5 g samples were acid digested with a 3:1 mixture of HNO_3_ and HClO_4_ for 6 h at ambient temperature and 12 h at 95 °C [51]. The samples were centrifuged for 10 min at 10,000× *g*. Following supernatant extraction, the samples were diluted to 10 mL with deionized water to allow the analytical instruments to quantify metals. Atomic absorption spectroscopy (Perkin-Elmer Analyst 400, was used to determine the amount of lead in water and plant samples. Treatments were replicated three times. Lead concentration values were expressed as mg/L (ppm) for water samples and mg/kg DW for plant samples.

The efficiency of phytoremediation in polluted water is calculated using the metal removal proportion. The next equation was used to calculate it [52]:

(1) $$\%\;\mathrm{Removal}\;\mathrm{efficiency}=\frac{\left(\mathrm{Ci}\;-\;\mathrm{Cf}\right)\;}{\mathrm{Ci}}\;\;\times 100\;$$

where Ci is the water’s initial metal concentration and Cf is the water’s final metal concentration.

The bioconcentration factor (BCF) is an important measure that indicates a plant’s ability to accumulate metals in relation to the metal concentration in the medium [53]. The BCF formula is given as follows:

BCF = CP/CW(2)

CP indicates the trace element concentration in plant tissue (mg/kg) and CW represents the trace element concentration in water (mg/L).

The transfer factor (TF) relates to the plant’s internal metal transport and is defined as the movement of heavy metal from the roots to the aerial section. A ratio of metal accumulated in the shoot to metal accumulated in the root is used to calculate the translocation factor [54].

TF = Cl/Cr(3)

where Cl represents the metal concentration in leaf tissues (mg/kg) and Cr represents the metal concentration in roots (mg/kg). A higher TF value indicates greater translocation capability. TF > 1 implies that the plant effectively transports metals from the root to the shoot.

### 4.3. Determination of Photosynthetic Pigments

Chlorophyll *a*, chlorophyll *b*, and carotenoids were extracted in 85% acetone from the leaf samples, according to the method recommended by [55] Pigments extract were filtrated and the content of Chl. *a*, Chl. *b* and carotenoids were determined with spectrophotometry at the wavelengths of 663, 647, and 470 nm.

### 4.4. Antioxidant Enzymes Activity

One gram of plant tissue was homogenized in 1 mL K-phosphate buffer pH 7 and 1% PVP, and then centrifuged at 15,000 rpm for 15 min at 4 °C. The enzyme assay was performed on the supernatant. The activity of superoxide dismutase (SOD) was measured using the method described in [56]. The activity of ascorbate peroxidase (APX) was measured using the method described in [38,57]. The activity of guaiacol peroxidase (GPOD) was measured according to [58].

### 4.5. Fresh and Dry Weight

Plants exposed to lead at various times and concentrations, as well as plants in the control group, were weighed after draining water carefully with filter papers to evaluate fresh weights. These plants were instantly frozen and kept at −30 °C for biochemical analysis. To determine the constant weight for dry mass estimation, some parts of the samples were rapidly dried in an oven at 70 °C for 48 h. 

### 4.6. Determination of Organic Solutes

According to the method of [59], soluble proteins content was determined. Soluble sugars content was estimated according to [60]; the soluble carbohydrate concentration was measured in mg/g FW.

### 4.7. Statistical Analysis

Software package of MINITAB 17.0 for Windows was performed to the statistical analyses of the results including: mean, SD, Pearson correlation coefficients, etc. for the measured data (*n* = 3). One-way analysis of variance (ANOVA) was performed for all parameters and then the significance of differences between mean values for each treatment was determined according to Tukey’s multiple comparison tests at *p* < 0.05.

## 5. Conclusions

*Ludwigia stolonifera* (Guill. & Perr.) has been shown in this study to have a high capacity for extracting Pb from the surrounding water, with BCFs exceeding 1000. When a plant has the potential to bioconcentrate an element in its tissues, it is considered a strong accumulator of metals. Lead stress alters a range of physiological parameters, including a decrease in chlorophyll content, protein, soluble carbohydrate, and a rise in antioxidative enzyme activities such as SOD, APX, and POD. According to the findings, *L. stoloniferia* is appropriate for phytoremediation of Pb contamination in water at low and high lead concentrations. As a result, *L. stoloniferia* may be useful for removing Pb from polluted water through phytoremediation.

## Figures and Tables

**Table 1:** Residual lead concentration in solution at different exposure times.

Concentration (mg/L)	Pb Concentration in Solution (mg/L)			
	1 d	3 d	7 d	10 d
0	ND	ND	ND	ND
10	2.67 ^a^ ± 0.04	1.27 ^b^ ± 0.28	0.59 ^c^ ± 0.13	0.44 ^c^ ± 0.04
25	8.11 ^a^ ± 0.75	3.98 ^b^ ± 0.58	2.31 ^bc^ ± 0.14	1.84 ^c^ ± 0.95
50	33.09 ^a^ ± 4.40	17.96 ^b^ ± 1.33	7.23 ^c^ ± 0.41	4.14 ^c^ ± 0.70
100	43.9 ^a^ ± 4.61	26.43 ^b^ ± 0.64	11.99 ^c^ ± 0.78	9.92 ^c^ ± 0.92

ND = Not Detected.; d = day. Data are means ± SE, *n* = 3. Different letters in row indicate significant differences between treatments at *p* < 0.05 according to Tukey test.

**Table 2:** The removal efficiency of lead. by *L. Stolonifera* in different concentrations of lead for different exposure times.

Concentration (mg/L)	Pb Removal Efficiency (%)			
	1 d	3 d	7 d	10 d
0	ND	ND	ND	ND
10	73.28	87.22	94.06	95.58
25	67.53	84.06	90.76	92.61
50	33.81	64.06	85.53	91.71
100	56.11	73.56	88.01	90.08

ND = Not Detected; d = day.

**Table 3:** Lead accumulation in *Ludwigia stolonifera* treated with different concentration of lead for different exposure times.

Concentration (mg/L)	Pb Accumulation (mg/kgDW)			
	1 d	3 d	7 d	10 d
0	ND	ND	ND	ND
10	320.33 ^c^ ± 34.26	665.75 ^b^ ± 18.27	834.33 ^ab^ ± 87.58	875 ^a^ ± 90.13
25	866.66 ^c^ ± 105.97	1668 ^b^ ± 165.5	2543.16 ^a^ ± 265.92	2681.67 ^a^ ± 408.36
50	1871.75 ^c^ ± 294.20	3140.5 ^b^ ± 453.44	4725 ^a^ ± 270.41	4975 ^a^ ± 340.18
100	3963.3 ^c^ ± 509.24	5078.7 ^b^ ± 153.98	6540 ^a^ ± 51.96	6840 ^a^ ± 90

ND = Not Detected; d = day. Data are means ± SE, *n* = 3. Different letters indicate significant differences between treatments at *p* < 0.05 according to Tukey test.

**Table 4:** Bioconcentration factor (BCF), and translocation factor (TF) for lead in *Ludwigia stolonifera*.

Concentration (mg/L)	(BCF)				(TF)			
	1 d	3 d	7 d	10 d	1 d	3 d	7 d	10 d
10	119.90	520.79	1406.17	1981.13	1.24	1.11	1.16	1.17
25	106.77	418.74	1426.07	1452.16	0.92	1.08	1.16	1.16
50	56.55	174.79	653.22	1199.75	1.27	1.41	1.48	1.49
100	90.28	192.13	530.11	689.51	1.53	1.58	1.58	1.85

d = day.

**Table 5:** Summary statistics, regression analysis, and correlations of the studied parameters under the effects of exposure time (t) and metals concentration (c). *: *p* < 0.05, **: *p* < 0.01, ***: *p* < 0.001.

	Person’s Correlation		Regression Analysis			
	(c)	(t)	R^2^ (c) (%)	R^2^ (t) (%)	Equation Model (c)	Equation Model (t)
**Chlorophyll a**	−0.627 ***	−0.677 ***	39.9	45.9	y = 1134−0.3032x	y = 1198−34.62x
					*p* < 0.001, r = −0.63	*p* < 0.001, r = −0.68
**Chlorophyll b**	−0.536 ***	−0.588 ***	28.7	34.6	y = 647.5−0.3314x	y = 720.7−38.44x
					*p* < 0.001, r = −0.54	*p* < 0.001, r = −0.60
**Carotenoid**	0.357 *	0.868 ***	12.8	75.3	y = 559.2 + 0.3426x	y = 194.0 + 87.98x
					*p* < 0.05, r = 0.36	*p* < 0.001, r = 0.86
**Total chlorophyll**	−0.590 ***	−0.664 ***	34.75	44.1	y = 1759.2–6.04x	y = 1829.3–66.6x
					*p* < 0.001, r = −0.58	*p* < 0.001, r = −0.66
**Chl a/ Chl b**	0.176	0.563 ***	3.11	31.7	y = 2.136 + 0.0049x	y = 1.551 + 0.1553x
					*p* < 0.001, r = 0.17	*p* < 0.001, r = 0.56
**APX**	0.546 ***	0.540 ***	29.9	29.1	y = 4.187 + 0.0039x	y = 3.593 + 0.4087x
					*p* < 0.001, r = 0.55	*p* < 0.001, r = 0.50
**POD**	0.407 **	0.786 ***	16.5	56.8	y = 1284 + 0.830x	y = 661.1 + 169.6x
					*p* < 0.01, r = 0.41	*p* < 0.001, r = 0.75
**SOD**	0.841 ***	0.461 ***	70.7	21.3	y = 20.17 + 0.0808x	y = 30.42 + 4.689x
					*p* <0.001, r = 0.84	*p* < 0.01, r = 0.47
**Fresh weight**	−0.578 ***	0.378 **	33.39	14.27	y = 2.681−0.0104x	y = 1.853 + 0.0663x
					*p* < 0.001, r = −0.57	*p* < 0.001, r = 0.37
**Dry weight**	−0.758 ***	0.276	57.48	7.61	y = 37.02−0.199x	y = 24.10 + 0.708x
					*p* < 0.001, r = −0.76	*p* < 0.001, r = 0.27
**Protein**	0.273	−0.731 ***	7.45	53.43	y = 8.188 + 0.0135x	y = 10.677–0.354x
					*p* < 0.001, r = 0.27	*p* < 0.001, r = −0.73
**Carbohydrate**	0.287	−0.687 ***	6.87	45.78	y = 54.67 + 0.0221x	y = 70.877–0.954x
					*p* < 0.001, r = 0.36	*p* < 0.001, r = −0.65

## Data Availability

The datasets generated and/or analyzed during the current study are available from the corresponding author upon reasonable request.
